# Comment on “Global clinical trial landscape of microbiome modulator therapy in sepsis: gut microbiota interventions and challenges”

**DOI:** 10.1097/JS9.0000000000005074

**Published:** 2026-03-12

**Authors:** Guohua You, Yiran Ren, Yuming Du

**Affiliations:** Comprehensive ICU Ward (2), First Affiliated Hospital of Zhengzhou University, Zhengzhou, Henan, China

*To the Editor,*

We read with great interest the letter by Ma *et al*^[^1^]^ on the global clinical trial landscape of microbiome-based therapies for sepsis. This paper provides a comprehensive overview of 112 trials up to 2025, highlighting the prominence of probiotics (61%) and fecal microbiota transplantation (FMT, 12%) as the main interventions, as well as the geographic focus on North America, Europe, and the Asia-Pacific region. The analysis underscores the growing recognition of gut dysbiosis as a key factor in the pathogenesis of sepsis, and microbiome-targeted therapies aimed at restoring balance and alleviating immune dysregulation. However, while we acknowledge the value of their contribution, we believe that the study warrants further discussion and refinement in the following areas:

## Methodological limitations

The study relied solely on data from the Trialtrove database, which may introduce significant selection bias due to the omission of trials registered in comprehensive platforms such as ClinicalTrials.gov or the EU Clinical Trials Register. Multiple studies have shown that a comprehensive systematic review should integrate data from multiple registry platforms. For instance, Zarin *et al*^[^2^]^ in *The Lancet* demonstrated significant data discrepancies between ClinicalTrials.gov and the WHO International Clinical Trials Registry Platform. Furthermore, a meta-analysis of 37 randomized controlled trials (RCTs) on probiotics in critically ill patients found no mortality benefit^[^3^]^. This single data source may lead to an incomplete description of the trial landscape, particularly by overlooking early-phase or negative-result studies.

## Insufficient safety assessment

Although the study highlights the therapeutic potential of probiotics and FMT, the discussion of safety risks is inadequate. For example, bacteremia, endocarditis, and sepsis related to probiotics such as *Lactobacillus rhamnosus*, as reported in cases involving cardiac surgery and neonatal patients^[^4,5^]^. A matched case-control study further showed that hospitalized adults receiving probiotics had an increased risk of invasive infections originating from probiotic organisms (odds ratio = 2.3, 95% CI: 1.4–3.8; PMID: 34612183), underscoring the need for caution in high-risk populations^[^6^]^. Similarly, fecal microbiota transplantation poses a risk of transmitting multidrug-resistant organisms, as evidenced by an Food and Drug Administration alert following fatal cases^[^7^]^.

## Lack of methodological quality assessment

The study did not assess the methodological quality of the included trials. According to the Cochrane risk of bias assessment tool developed by Higgins *et al*^[^8^]^, significant differences in the quality of randomized trials may substantially impact the reliability of the results. The lack of quality assessment makes it difficult to determine the evidence level of the study’s findings.

## Recommendations

Overall, the work of Ma *et al*^[^1^]^ effectively highlights the therapeutic potential of microbiome modulation in sepsis and lays the groundwork for clinical translation. However, addressing these limitations by incorporating broader data sources, discussing risks more thoroughly, and integrating meta-analysis evidence would strengthen future analyses. Prioritizing multicenter RCTs, carefully considering risks and antibiotic effects in different populations, will be crucial to realizing these benefits.

## Data Availability

No new data were generated or analyzed for this correspondence.

## AI assistance

A generative AI-based language model was employed solely for language polishing.
